# Supplementary material for: The interactome of intact mitochondria by cross-linking mass spectrometry provides evidence for coexisting respiratory supercomplexes
Source: Mol Cell Proteomics. 2017 Dec 8;17(2):216–32. doi: 10.1074/mcp.RA117.000470 (PMC5795388; doi:10.1074/mcp.RA117.000470)
Supplement: Supplemental Data [file supp_RA117.000470_133922_0_supp_23978_fzffwf.zip › spectra_annotation/mito_DR_spectra_annotation/180-1-2-1-8-1.pdf]

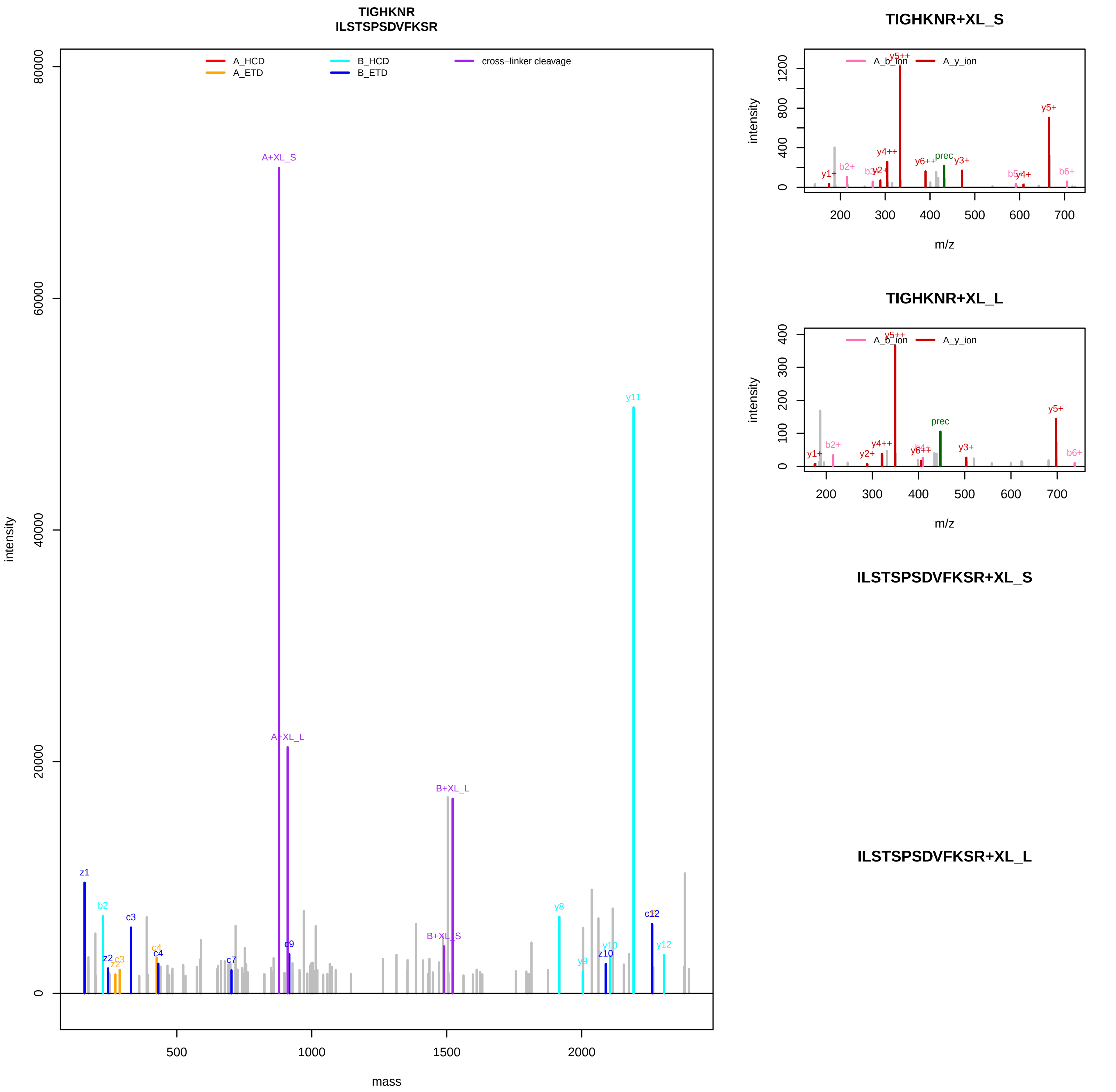
TIGHKNR
ILSTSPSDVFKSR
A_HCD
A_ETD
B_HCD
B_ETD
cross−linker cleavage
A+XL_S
A+XL_L
B+XL_L
B+XL_S
y11
z1
b2
c3
z2
c3
z2
c4
c4
c7
c9
y8
y9
y10
z10
c12
y12
intensity
mass
0
20000
40000
60000
80000
500
1000
1500
2000
TIGHKNR+XL_S
A_b_ion
A_y_ion
y5++
y5+
y4++
y6++
prec
y3+
y1+
b2+
b3
y2+
b5
y4+
b6+
intensity
m/z
TIGHKNR+XL_L
A_b_ion
A_y_ion
y5++
y5+
y4++
y6++
prec
y3+
y1+
b2+
y2+
b4+
b6+
intensity
m/z
ILSTSPSDVFKSR+XL_S
ILSTSPSDVFKSR+XL_L
